# Integrating the lymphocyte-albumin score into prognostic stratification of nasopharyngeal carcinoma patients treated with concurrent chemoradiotherapy

**DOI:** 10.3389/fphys.2026.1813058

**Published:** 2026-07-08

**Authors:** Wei-Qiong Ni, Fei Xu, Hai-Ping You, Xu-Xin Lin, Yong-Miao Lin, Xin Huang, Wen Xia, Yu-Ling Zhang, Sha-Sha Du, Xin Hua

**Affiliations:** 1 Department of Radiation Oncology, Shanghai Jiao Tong University Medical School Affiliated Ruijin Hospital, Shanghai, China; 2 Department of Radiation Oncology, Guangdong Provincial People’s Hospital, Guangdong Academy of Medical Sciences, Southern Medical University, Guangzhou, China; 3 School of Medicine South China University of Technology, Guangzhou, China; 4 Sun Yat-sen University Cancer Center, State Key Laboratory of Oncology in South China, Guangdong Provincial Clinical Research Center for Cancer, Guangzhou, China; 5 Guangdong Key Laboratory of Nasopharyngeal Carcinoma Diagnosis and Therapy, Guangzhou, China; 6 Department of Endocrinology, Jiangxi Provincial People’s Hospital, The First Affiliated Hospital of Nanchang Medical College, Nanchang, China

**Keywords:** concurrent chemoradiotherapy, lymphocyte-albumin score, nasopharyngeal carcinoma, nomogram, prognostic factor

## Abstract

**Background:**

Emerging evidence indicates that the lymphocyte-albumin (LA) score, a novel biomarker that reflects systemic inflammation and nutritional status, has a strong connection with survival outcomes in a range of tumors; though its prognostic relevance in nasopharyngeal carcinoma (NPC) has not been extensively studied. The aim of this study is to explore the relationship between the LA score and survival outcomes in NPC and to establish a predictive model accordingly.

**Methods:**

This single-center retrospective study included 861 non-metastatic NPC patients who were treated with concurrent chemoradiotherapy (CCRT) between January 2010 and December 2014.Clinicopathological data, including the LA score, were collected for analysis. The optimal cutoff value for the LA score was calculated using maximally selected rank statistics. Both univariate and multivariate Cox regression analyses were conducted to identify independent prognostic factors. Additionally, a nomogram incorporating the LA score and other significant variables was developed to predict overall survival (OS).

**Results:**

Patients with elevated LA scores (>70.56) demonstrated significantly improved OS compared to those with lower scores (≤70.56) (HR = 0.572, 95% CI: 0.424-0.771, P<0.001). The multivariate analysis revealed that the LA score, along with age, T stage, N stage, and BMI, served as independent prognostic factors for OS. During a median follow-up of 123.2 months, 174 deaths were recorded. The nomogram incorporating these variables demonstrated moderate discriminative ability (C-index = 0.671, 95% CI: 0.631–0.710) and showed modest improvement over the conventional TNM staging system.

**Conclusion:**

This study is the first to highlight the prognostic relevance of the LA score in NPC patients undergoing CCRT. The LA score, being a straightforward and readily available biomarker, can complement anatomical staging for better risk stratification and personalized treatment strategies in this challenging malignancy.

## 1 Introduction

Nasopharyngeal carcinoma (NPC) is considered relatively uncommon globally, yet it remains endemic in East and Southeast Asia, contributing to around 130,000 new cases each year (Sung et al., 2021). Over 70% of these cases are diagnosed at a locoregionally advanced stage (Mao et al., 2009; Zhang et al., 2023b), for which concurrent chemoradiotherapy (CCRT) is the prevailing treatment method (Langendijk et al., 2004; Chen et al., 2019). Although the tumor-node-metastasis (TNM) staging system provides essential guidance for outcome predictions and treatment decisions (Amin et al., 2017; Chen et al., 2021; Tang et al., 2021), there exists substantial interpatient variability in survival rates, even among individuals sharing identical TNM stages (Amin et al., 2017; Hui et al., 2020). Studies suggest that approximately 30% of patients may experience disease progression despite receiving comparable therapeutic approaches, highlighting the limitations of TNM staging in capturing the biological complexity of NPC (Lee et al., 2015; Zhang et al., 2016). This variability accentuates the urgent necessity for the identification of novel prognostic biomarkers capable of enhancing anatomical staging and optimizing treatment, ultimately contributing to improved clinical outcomes in patients afflicted with this aggressive cancer (Gradel, 2023).

While multiple inflammatory biomarkers such as the neutrophil-to-lymphocyte ratio (NLR) and platelet-to-lymphocyte ratio (PLR) have been investigated in NPC, these markers primarily reflect the balance between pro-inflammatory and anti-inflammatory cell populations (Takenaka et al., 2017). In contrast, the LA score integrates two complementary aspects of the host response: immune competence (lymphocyte count) and nutritional status (albumin level). This dual integration provides a more comprehensive assessment of the systemic tumor-host interaction compared to single-cell ratio markers. Additionally, unlike NLR and PLR, which can be acutely affected by transient inflammatory events, albumin levels reflect longer-term nutritional and inflammatory status, making the LA score a more stable prognostic indicator.

The LA score has demonstrated prognostic value in various solid tumors, including colorectal cancer, gastric cancer, and hepatocellular carcinoma (Yamamoto et al., 2021; Zhang et al., 2023). However, these studies have primarily focused on patients undergoing surgical resection, and its role in patients receiving definitive chemoradiotherapy remains understudied (Miyata et al., 2024). Specifically, no large-scale study has systematically evaluated the prognostic significance of the LA score in NPC—a malignancy highly dependent on chemoradiotherapy and characterized by a unique inflammatory tumor microenvironment driven by Epstein-Barr virus (EBV) infection. This represents a critical gap in the literature, as systemic inflammation and nutritional status are particularly important determinants of treatment response and survival in patients receiving CCRT.

Recent studies emphasize the critical involvement of systemic inflammation in cancerogenesis and prognosis. Both inflammatory and nutritional conditions play a significant role in determining treatment sensitivity and overall outcomes (Hanahan and Weinberg, 2011; Diakos et al., 2014; Zitvogel et al., 2017). Key hematological parameters, such as albumin and lymphocyte counts, have emerged as promising biomarkers for evaluating these conditions (Ménétrier-Caux et al., 2019; Chen et al., 2020; Kuang et al., 2024). Albumin has been established as an important prognostic indicator across a multitude of malignancies, including hepatocellular carcinoma and non-small cell lung cancer (Jeng et al., 2023; Zhang et al., 2023a; Zhen et al., 2024), while lymphocyte levels increasingly reflect immune functionality (Zhen et al., 2024). In this regard, the lymphocyte×albumin (LA) score—a novel integrative biomarker—has shown potential in predicting survival outcomes in metastatic colorectal cancer and various solid tumors, indicating the balance between inflammatory states and immune responses (Yamamoto et al., 2021; Miyata et al., 2024; Okazaki et al., 2025). Despite the acknowledgment of numerous inflammatory markers, including the platelet-to-lymphocyte ratio (PLR) and neutrophil-to-lymphocyte ratio (NLR) (Chua et al., 2016; Sun et al., 2016; Jiang et al., 2017; Zhao et al., 2022), the specific prognostic significance of the LA score in NPC has not been comprehensively evaluated. Given the critical role of inflammation within the tumor microenvironment and its implications for treatment responsiveness, there is a significant opportunity to explore the prognostic implications of the LA score for patients with NPC undergoing CCRT.

To bridge this gap, our study aims to elucidate the prognostic significance of the LA score in NPC patients undergoing CCRT through a large-scale retrospective analysis. By clarifying the relationship between the LA score and patient outcomes, we seek to contribute to the existing knowledge base surrounding inflammatory markers in NPC. Furthermore, we plan to develop a predictive nomogram that utilizes LA score values for individual survival predictions, effectively integrating the multifaceted roles of inflammation and immune response in cancer prognosis. This nomogram aspires to enhance the currently available prognostic tools, potentially improving prognostic stratification and supporting future individualized treatment strategies for this challenging malignancy, ultimately fostering improved therapeutic strategies and patient outcomes.

## 2 Methods

### 2.1 Patients

This large-scale, long-term retrospective study enrolled consecutive patients diagnosed with non-metastatic NPC who received platinum-based CCRT at the Sun Yat-sen University Cancer Center (SYSUCC) during the period from January 2010 to December 2014. Inclusion criteria were as follows: (i) treatment-naïve NPC confirmed by histological and radiographic assessments; (ii) availability of pre-treatment tests of peripheral blood and Epstein-Barr virus (EBV) DNA; (iii) receipt of radical intensity-modulated radiotherapy combined with weekly or triweekly platinum-based concurrent chemotherapy; and (iv) no history of chronic inflammatory disease, active infection, severe liver dysfunction, severe renal disease, autoimmune disease requiring immunosuppressive therapy, long-term corticosteroid use, or other conditions that could substantially influence serum albumin levels or peripheral lymphocyte counts. Complete data for all variables included in the final analysis were available for all 861 patients. Patients with missing pre-treatment laboratory data (n=65) were excluded from the study cohort prior to analysis. All patients were staged according to the 8th edition of the American Joint Committee on Cancer (AJCC) TNM system. The Institutional Review Board of SYSUCC approved this retrospective study and waived the requirement for written informed consent, as the research was non-interventional and adhered to all relevant ethical guidelines.

### 2.2 Data acquisition and follow-up procedures

Baseline laboratory and clinicopathological data were collected from medical records within one week of diagnosis. Plasma EBV-DNA levels (copies/ml) were quantified using real-time quantitative polymerase chain reaction methods. Body mass index (BMI) was calculated using weight (kg) divided by height squared (m^2^), classifying patients into obese (BMI≥28 kg/m^2^), overweight (24<BMI < 28 kg/m^2^), or non-obese (BMI ≤ 24 kg/m^2^). The LA score was calculated as the product of the absolute lymphocyte count (10^9^/L) and serum albumin level (g/L). All patients received radical intensity-modulated radiotherapy (IMRT) combined with platinum-based concurrent chemotherapy. Two concurrent chemotherapy regimens were used: (1) weekly cisplatin at a dose of 30–40 mg/m^2^ for 6–7 cycles; (2) triweekly cisplatin at a dose of 80–100 mg/m^2^ for 2–3 cycles. Among the 861 patients, 649 (75.4%) received triweekly cisplatin and 212 (24.6%) received weekly cisplatin. No patients received induction chemotherapy or adjuvant chemotherapy as part of their initial treatment. Treatment and follow-up protocols conformed to previously established guidelines. Overall survival (OS) was defined as the interval from the date of diagnosis to either the date of death or the last follow-up.

### 2.3 Statistical analysis

Numerical variables were presented as medians and interquartile ranges (IQR), whereas categorical variables were expressed as counts and percentages. The optimal cutoff value for the LA score was calculated using maximally selected rank statistics with survival status as the endpoint. Kaplan-Meier curves were generated, with log-rank tests employed for comparisons. Univariate and multivariate Cox regression analyses were conducted to identify independent prognostic variables, and significant prognostic factors were utilized to construct a nomogram. Harrell’s concordance index (C-index), time-dependent receiver operating characteristic (tROC) analysis, and decision curve analysis (DCA) were used to assess the discriminative ability and clinical utility of the prognostic model in the study cohort. A two-tailed P-value < 0.05 was regarded as statistically significant. All statistical analyses were performed using R version 4.2.1.

Prior to multivariable Cox regression analysis, multicollinearity among candidate variables was assessed using variance inflation factors (VIFs). A VIF value >5 was considered indicative of significant multicollinearity.

## 3 Results

### 3.1 Characteristics of the patients

**Figure 1:**
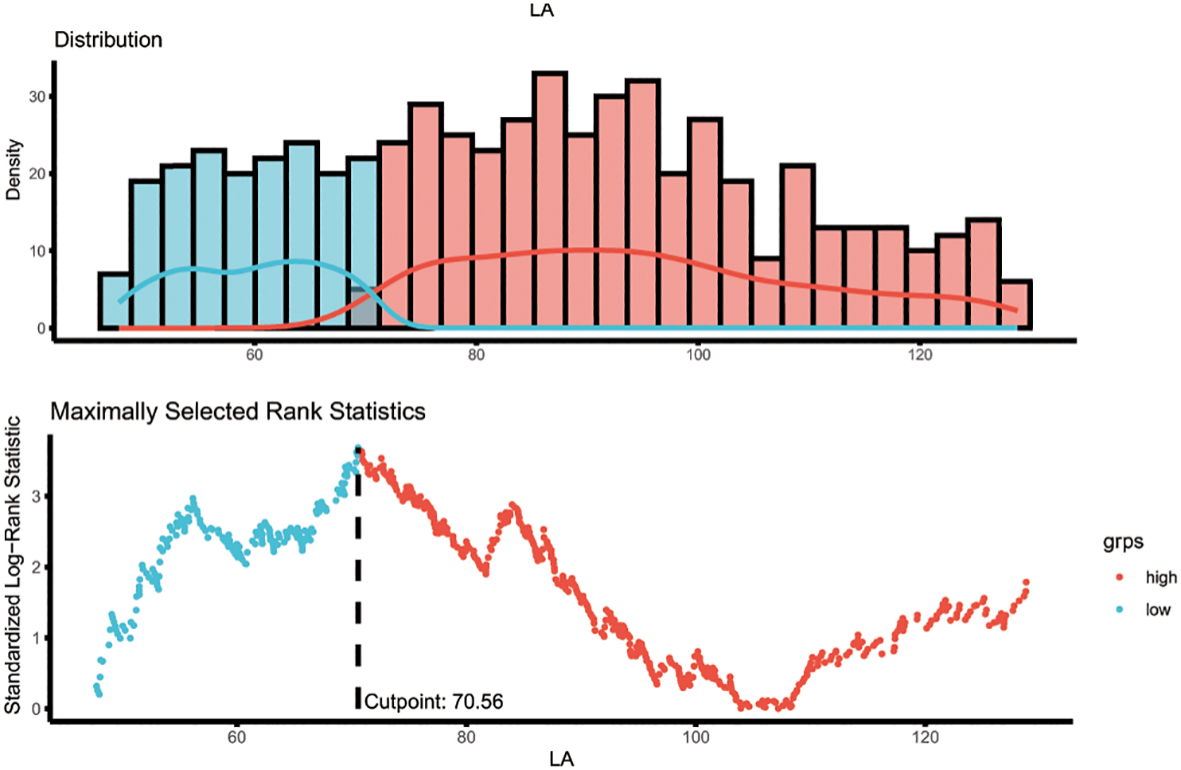
Derivation of the cutoff value of the LA score according to maximally selected log-rank statistics.

In total, 861 NPC patients were enrolled in the present study. An overview of the clinicopathological features at baseline is provided in **Table 1**. Among the patients, 221 (25.7%) were female and 640 (74.3%) were male; with a median age of 45 years (range: 18–84 years). Out of the participants, 423 (49.1%) were over 45 years old, while 438 (50.9%) were under this age. The majority were diagnosed with histological type WHO III, and 283 (32.9%) exhibited EBV-DNA levels ≥ 4000 copies/ml. Based on maximally selected rank statistics, patients were classified into two groups according to their LA values: high-LA (> 70.56, n = 585) and low-LA (≤ 70.56, n = 276) (**Figure 1**).

**Table 1:** Patient demographics and clinical characteristics.

Characteristic	No. (%) of patients
Age	
>45 years	423 (49.1%)
≤45 years	438 (50.9%)
Gender	
Male	640 (74.3%)
Female	221 (25.7%)
Histology group	
WHO I/II	13 (1.51%)
WHO III	848 (98.5%)
HGB group	
<113 g/L	27 (3.14%)
113–151 g/L	548 (63.6%)
≥151 g/L	286 (33.2%)
LDH group	
≥245U/L	51 (5.92%)
<245 U/L	810 (94.1%)
T stage	
T1	42 (4.88%)
T2	165 (19.2%)
T3	524 (60.9%)
T4	130 (15.1%)
N stage	
N0	81 (9.41%)
N1	465 (54.0%)
N2	271 (31.5%)
N3	44 (5.11%)
BMI group	
≤24 kg/m^2^	519 (60.3%)
24–28 kg/m^2^	294 (34.1%)
≥28 kg/m^2^	48 (5.57%)
EBV-DNA group	
<4,000 copies/ml	578 (67.1%)
≥4,000 copies/ml	283 (32.9%)
LA group	
>70.56	585 (67.9%)
≤70.56	276 (32.1%)
Chemotherapy regimen	
weekly cisplatin	649 (75.4%)
triweekly cisplatin	212 (24.6%)
KPS	
80	3 (0.35%)
90	858 (99.7%)

WHO, World Health Organization; HGB, hemoglobin; LDH, serum lactate dehydrogenase levels; BMI, body mass index; EBV-DNA, Epstein-Barr virus DNA; LA, lymphocyte-albumin.

### 3.2 Prognostic significance of LA in NPC

**Figure 2:**
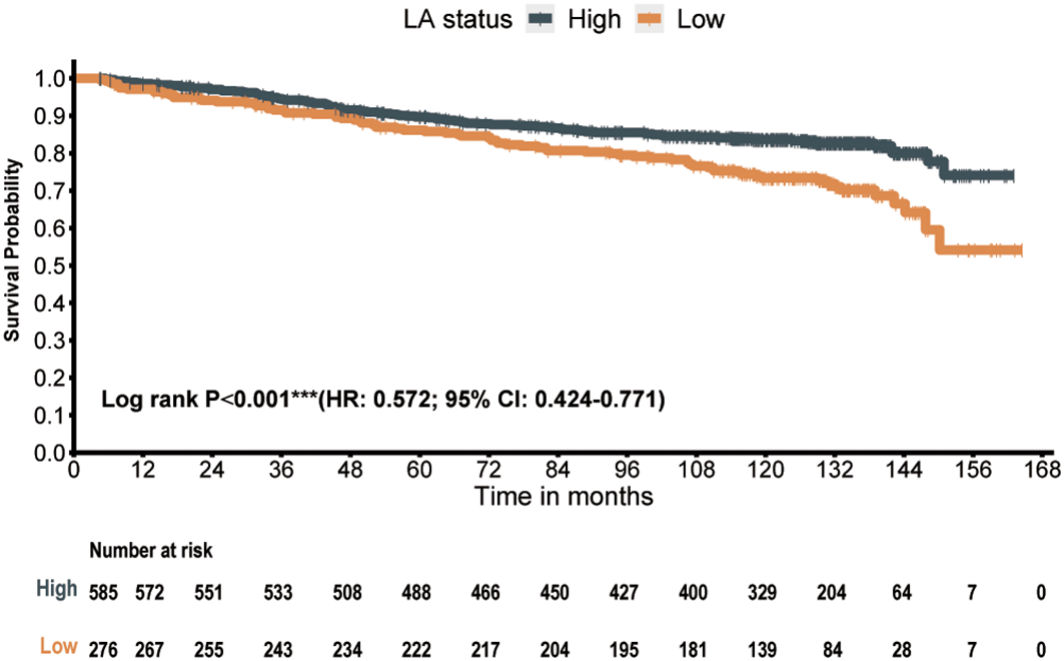
Survival curves obtained with Kaplan-Meier analysis between different LA Groups (the HRs reported were unadjusted). LA, lymphocyte-albumin; HR, hazard ratios; CI, confidence interval.

The median OS among patients was 123.2 months (IQR: 87.8-136.0 months). During the study period, 174 events were recorded. The OS rates for 1-, 3-, 5-, and 10-year were 98.1%, 93.6%, 88.6%, and 80.3%, respectively. Survival analysis indicated that patients with high LA scores experienced significantly better overall survival compared to those with low LA scores (**Figure 2**, HR: 0.572; 95% CI: 0.424-0.771, P < 0.001).

### 3.3 Cox regression analyses of OS in NPC

**Figure 3:**
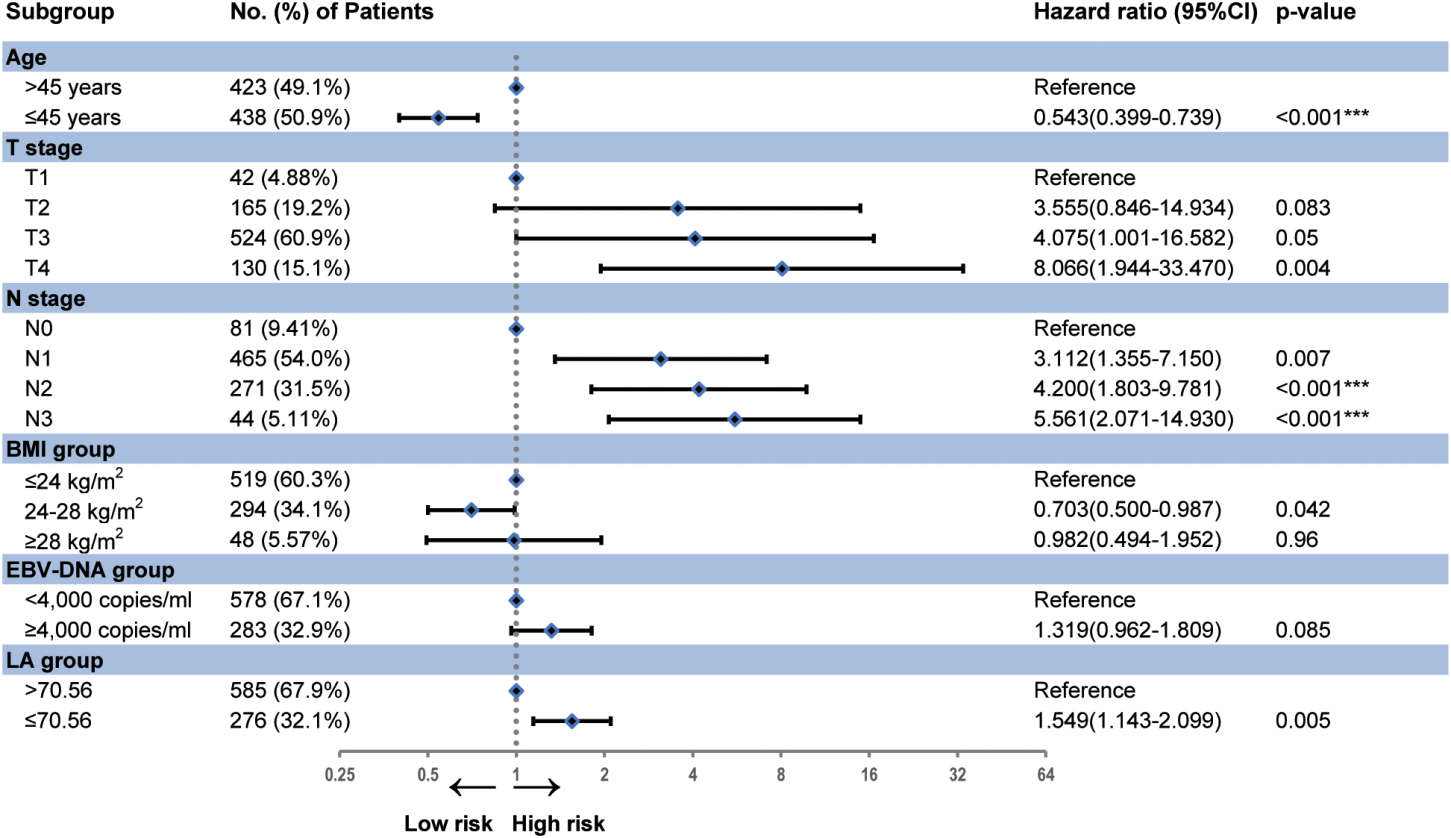
Multivariable analysis for overall survival of nasopharyngeal carcinoma patients.

In the univariate analysis, several factors showed significant associations with OS: age (≤ 45 years, HR = 0.567, 95% CI: 0.418-0.769, P < 0.001), T stage (T4 vs T1: HR = 9.208, 95% CI: 2.232-37.990, P = 0.002; T3 vs T1: HR = 4.494, P = 0.035), N stage, BMI (24–28 kg/m^2^ vs ≤ 24 kg/m^2^: HR = 0.713, 95% CI: 0.511-0.996, P = 0.047), EBV-DNA (≥ 4000 copies/mL: HR = 1.686, 95% CI: 1.248-2.277, P < 0.001), and LA score. Nutritional support during treatment was provided at the discretion of the treating physician, with oral nutritional supplements recommended for patients with weight loss >5% or BMI <18.5 kg/m^2^. These variables were evaluated as potential confounders in univariate analysis but were not included in the final multivariate model due to lack of statistical significance (all p>0.05). The multivariate analysis identified five independent prognostic factors: age (≤ 45 years: HR = 0.543, 95% CI: 0.399-0.739, P < 0.001); T stage (T4 vs T1: HR = 8.066, 95% CI: 1.944-33.470, P = 0.004); N stage (N1: HR = 3.112, 95% CI: 1.355-7.150, P = 0.007; N2: HR = 4.200, 95% CI: 1.803-9.781, P<0.001; N3: HR = 5.561, 95% CI: 2.071-14.930, P<0.001); BMI (24–28 kg/m^2^: HR = 0.703, 95% CI: 0.500-0.987, P = 0.042); and LA (≤ 70.56: HR = 1.549, 95% CI: 1.143-2.099, P = 0.005) (**Figure 3**). Notably, the significance of EBV-DNA status was diminished in the multivariate analysis (P = 0.085, **Table 2**).

**Table 2:** Univariate and multivariate Cox regression analyses of variables for overall survival in patients with non-metastatic NPC following platinum-based CCRT.

Characteristic	Univariate analysis		Multivariate analysis	
	Hazard ratio (95% CI)	p-value	Hazard ratio (95% CI)	p-value
Age				
>45 years	1		1	
≤45 years	0.567 (0.418-0.769)	<0.001***	0.543 (0.399-0.739)	<0.001***
Gender group				
Male	1			
Female	0.877 (0.620, 1.242)	0.461		
Histological type				
WHO I/II	1			
WHO III	0.449 (0.184-1.092)	0.077		
HGB group				
≤113 g/L	1			
113–151 g/L	1.423 (0.525-3.860)	0.488		
≥151 g/L	1.399 (0.507-3.856)	0.517		
LDH group				
≥245 U/L	1			
<245 U/L	0.626 (0.362-1.081)	0.093		
KPS				
80	1			
90	0.998 (0.990-1.007)	0.672		
T stage				
T1	1		1	
T2	4.118 (0.983-17.260)	0.053	3.555 (0.846-14.934)	0.083
T3	4.494 (1.108-18.220)	0.035	4.075 (1.001-16.582)	0.050
T4	9.208 (2.232-37.990)	0.002	8.066 (1.944-33.470)	0.004
N stage				
N0	1		1	
N1	2.763 (1.209-6.316)	0.016	3.112 (1.355-7.150)	0.007
N2	3.603 (1.561-8.318)	0.003	4.200 (1.803-9.781)	<0.001***
N3	5.010 (1.902-13.199)	0.001	5.561 (2.071-14.930)	<0.001***
BMI group				
≤24 kg/m^2^	1		1	
24–28 kg/m^2^	0.713 (0.511-0.996)	0.047	0.703 (0.500-0.987)	0.042
≥28 kg/m^2^	0.817 (0.414-1.610)	0.559	0.982 (0.494-1.952)	0.960
EBV-DNA group				
<4,000 copies/ml	1		1	
≥4,000 copies/ml	1.686 (1.248-2.277)	<0.001***	1.319 (0.962-1.809)	0.085
LA group				
>70.56	1		1	
≤70.56	1.749 (1.297-2.359)	<0.001***	1.549 (1.143-2.099)	0.005

Hazard ratios estimated by Cox proportional hazards regression. All statistical tests were two-sided. WHO, World Health Organization; HGB, hemoglobin; LDH, serum lactate dehydrogenase levels; BMI, body mass index; EBV-DNA, Epstein-Barr virus DNA; LA, lymphocyte-albumin.

***, P<0.001; **, P<0.01; *, P<0.05.

The proportional hazards assumption for the Cox regression model was verified using Schoenfeld residual tests (**Supplementary Figure 1**). No significant violation of the proportional hazard assumption was detected for any variable included in the final model (all P>0.05). In addition, Multicollinearity assessment demonstrated that all variables included in the final multivariable model had VIF values below 2, indicating no evidence of significant multicollinearity.

### 3.4 Construction of a prognostic model grounded in LA

**Figure 4:**
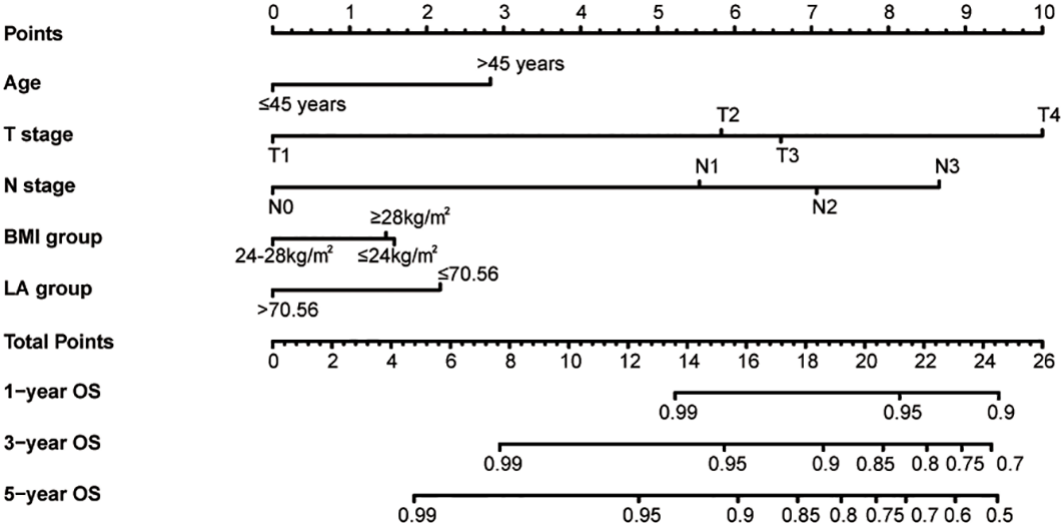
Presenting the nomogram for predicting 1-, 3-, and 5-year OS in NPC patients treated with CCRT. To use the nomogram, locate the patient’s value for each prognostic factor on the corresponding axis, draw a vertical line upward to the ‘Points’ axis to determine the points for each factor, sum the points for all factors, and then draw a vertical line downward from the ‘Total Points’ axis to the survival probability axes to obtain the predicted survival probabilities. BMI, body mass index; LA, lymphocyte-albumin; OS, overall survival.

Utilizing these independent prognostic factors, we constructed a nomogram that includes age, T stage, N stage, BMI, and the LA score to predict overall survival in NPC patients receiving CCRT (**Figure 4**). Each variable received a point value based on its relative contribution to prognosis as determined through multivariate Cox regression analysis. Prior to the initiation of CCRT, each patient’s total score is obtained by totaling the scores from each prognostic factor subclass, allowing for the estimation of 1-, 3-, 5-, and 10-year OS probabilities by mapping the total score onto the survival rate scale.

### 3.5 Evaluation and internal validation of the prognostic model’s predictive accuracy

**Figure 5:**
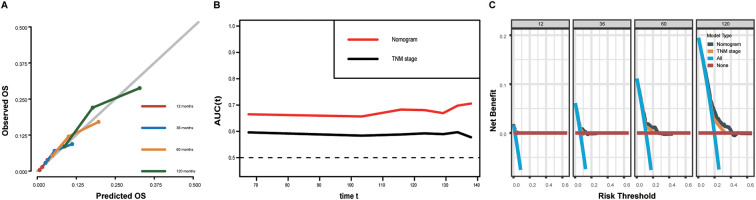
Assessment of predictive performance of the prognostic model. **(A)** Calibration plot of the nomogram model at 1, 3, and 5 years. **(B)** Time-dependent ROC curves compared the predictive accuracy of the current model and the traditional TNM stage. **(C)** DCA curves compared the net benefit rate of the current model and the traditional TNM stage. OS, overall survival; AUC, area under the curve; TNM, tumor node metastasis.

The prognostic model exhibited moderate discriminative ability, achieving a C-index of 0.671 (95% CI: 0.631-0.710), compared to a C-index of 0.638 (95% CI: 0.573-0.703) for the conventional TNM staging system. Calibration plots (with observed survival represented on the Y-axis and nomogram-predicted survival on the X-axis) for the 1-, 3-, 5-, and 10-year OS displayed strong agreement between predicted and observed outcomes. The prognostic accuracy of this model for customized OS was assessed via time-dependent ROC curves, demonstrating superiority over traditional TNM staging (**Figure 5**).

Internal validation using 1000 bootstrap resamples yielded a bootstrap-corrected C-index of 0.678 (95% CI: 0.637-0.717), indicating moderate stability of the prognostic model. The optimal LA score cutoff value of 70.56 was consistent across 91% of the bootstrap samples, suggesting reasonable robustness of the identified threshold.

### 3.6 Stratified analyses of the prognostic value of the LA score

Stratified analyses demonstrated that the prognostic value of the LA score remained consistent across different clinically relevant subgroups: TNM stage II (HR = 2.77, 95% CI:1.00-7.64, P = 0.049), TNM stage III (HR = 1.51, 95% CI: 1.02-2.23, P = 0.038), TNM stage IV (HR = 1.65, 95% CI: 0.97-2.81, P = 0.67), age ≤45 years (HR = 1.94, 95% CI: 1.20-2.41, P = 0.003), age >45 years (HR = 1.59, 95% CI: 1.08-2.32, P = 0.018), EBV-DNA <4000 copies/ml (HR = 1.68, 95% CI: 1.13-2.50, P = 0.011), and EBV-DNA ≥4000 copies/ml (HR = 1.71, 95% CI: 1.08-2.69, P = 0.022).

### 3.7 Interaction and incremental prognostic value analyses of the LA score

Interaction analysis revealed no significant interaction between the LA score and BMI (P for interaction = 0.682), suggesting that the prognostic effect of the LA score was consistent across BMI categories. Furthermore, the prognostic performance of the LA score was compared with its individual components. The LA score demonstrated superior discriminative ability (C-index = 0.623) compared with albumin alone (0.569, P = 0.018) and lymphocyte count alone (0.587, P = 0.032), indicating that the integration of nutritional and immune parameters may provide greater prognostic information than either component individually.

Based on the total nomogram score, patients were stratified into low-risk (total score ≤ 15) and high-risk groups (total score >15). Kaplan–Meier analysis demonstrated significantly poorer OS in the high-risk group than in the low-risk group (**Supplementary Figure 2**, P < 0.001).

## 4 Discussion

To our knowledge, this is the first large-scale study evaluating the prognostic significance of the LA score in NPC patients receiving CCRT. Our analysis indicated that those with elevated LA scores exhibited significantly better OS in comparison to those with lower scores. This finding reinforces the perspective that conventional anatomical staging systems, such as the TNM classification, may not sufficiently capture the variability in survival outcomes among NPC patients. The integration of the LA score within clinical practice could offer a more straightforward, non-invasive, and cost-effective means of improving prognostic evaluations in this diverse population.

NPC is characterized by significant biological heterogeneity, wherein patients at the same TNM stage can exhibit markedly different prognoses despite undergoing similar treatment regimens (Chiang et al., 2021). Advanced methodologies, including gene expression profiling (Tang et al., 2018) and liquid biopsies (Chan and Lo, 2002), have been explored in efforts to clarify the molecular complexities of NPC. However, many of these methods require considerable resources and are not readily implemented in routine clinical environments. Consequently, there is an urgent need for practical biomarkers capable of predicting individual patient outcomes independent of conventional classifications. Our findings indicate that the LA score, along with other indicators of nutritional and inflammatory status, could fulfill this role, contributing to the development of more personalized treatment approaches.

The correlation between inflammation, nutritional status, and survival outcomes in NPC patients further emphasizes the necessity of integrating various systemic markers into prognostic assessments (Fang et al., 2024). Our findings are consistent with past research that has established links between heightened systemic inflammation—as indicated by lower lymphocyte counts and albumin levels—and adverse prognostic outcomes. Elevated inflammation can disrupt immune regulation (Dolan et al., 2017), rendering individuals with lower LA values more susceptible to inadequate responses to CCRT and necessitating more tailored therapeutic interventions.

Elucidating the mechanisms that link the LA score to its prognostic significance could offer deeper insights into the interactions between immune function and tumor biology. Lymphocytes play a vital role in immune surveillance; their reduction due to malnutrition might considerably impair the body’s capacity to fight cancer (Ménétrier-Caux et al., 2019). Furthermore, albumin acts as an important surrogate marker for nutritional health, with hypoalbuminemia consistently associated with detrimental clinical outcomes across various malignancies (Li et al., 2018). Our findings therefore underscore the importance of monitoring immune function alongside nutritional status to effectively inform treatment choices.

Additionally, our analysis revealed age, N stage, and BMI as significant independent prognostic factors influencing OS in NPC patients. Age emerged as a particularly vital determinant, as older patients tend to be more prone to negative treatment effects and tend to exhibit worse survival rates. This observation corroborates previous research suggesting that age-related declines in immune function may worsen prognosis. Furthermore, the N stage has a critical role; advanced nodal involvement is closely correlated to nutritional deficits and poorer survival outcomes, supporting findings from Du et al. and Wei et al (Du et al., 2015; Wei et al., 2016).

Interestingly, although pretreatment EBV-DNA was significantly associated with OS in univariate analysis, it did not retain independent significance in the multivariable model. One possible explanation is that pretreatment EBV-DNA is closely associated with tumor burden and disease extent, and part of its prognostic effect may have been captured by TNM-related variables included in the model. In addition, emerging evidence suggests that dynamic EBV-DNA measurements obtained during or after treatment may provide stronger prognostic information than a single pretreatment assessment (Chan et al., 2017). Previous studies have also reported that pretreatment EBV-DNA may lose independent prognostic significance after adjustment for other clinicopathological factors (Lan et al., 2016; Hua et al., 2020). Furthermore, due to the retrospective nature of the historical data, we were unable to systematically adjust for certain potential confounders, such as cumulative smoking history and detailed comorbidity indices, owing to inconsistent documentation. However, given that 99.7% of our cohort had a KPS of 90 and predominantly EBV-driven WHO Type III disease, the confounding effects of these specific variables are likely minimized. Therefore, the loss of statistical significance observed in our study should be interpreted cautiously and does not diminish the established clinical importance of EBV-DNA in NPC.

The nomogram developed in this study represents a promising tool for prognostic stratification of NPC patients treated with CCRT. However, given the retrospective single-center design and lack of external validation, these findings should be considered exploratory and hypothesis-generating. Further prospective multicenter studies are required before this model can be implemented in routine clinical practice.

The prognostic significance of the LA score in NPC likely reflects the complex interplay between immune function, nutritional status, and the EBV-driven tumor microenvironment. EBV infection induces a chronic inflammatory state that can lead to lymphocyte exhaustion and malnutrition, both of which are associated with poor response to chemoradiotherapy. Lymphocytes are critical mediators of antitumor immunity, and their depletion impairs the ability of the immune system to eliminate residual tumor cells following treatment. Albumin, in addition to being a marker of nutritional status, has antioxidant and anti-inflammatory properties that may protect against treatment-related toxicity and enhance immune function. The combination of these two parameters therefore provides a more holistic assessment of the host’s ability to tolerate and respond to CCRT.

Our study has several important limitations that should be acknowledged. First, this was a retrospective single-center study conducted in an endemic Asian region, which may limit the generalizability of our findings to non-endemic regions and other ethnic populations. Second, the use of maximally selected rank statistics to derive the LA score cutoff value from the same cohort used for model development introduces a potential risk of overfitting. Third, we did not perform external validation in an independent cohort, which is essential to confirm the generalizability of the nomogram. Fourth, while we adjusted for several potential confounders, we did not have detailed information on dietary intake, muscle mass, or specific nutritional interventions, which may have influenced albumin levels and survival outcomes. Fifth, the moderate C-index of 0.671 indicates that the nomogram has only moderate discriminative ability, and further refinement with additional biomarkers is needed to improve its predictive performance. Finally, the retrospective design may have introduced selection bias, although we attempted to minimize this by including consecutive patients treated during the study period.

## 5 Conclusions

In conclusion, the LA score was identified as an independent prognostic factor for overall survival in NPC patients receiving CCRT. As a simple and readily available biomarker reflecting both immune and nutritional status, the LA score may provide prognostic information complementary to conventional TNM staging. Further multicenter prospective studies and external validation are warranted before clinical implementation.

## Data Availability

The data presented in this study are available on request from the corresponding author.

## Supplementary material

Supplementary Figure 1 Assessment of the proportional hazards assumption using Schoenfeld residuals for variables included in the multivariable Cox regression model.

Supplementary Figure 2 Kaplan–Meier curves for overall survival according to nomogram-based risk stratification.
