# Supplementary material for: Integrating the lymphocyte-albumin score into prognostic stratification of nasopharyngeal carcinoma patients treated with concurrent chemoradiotherapy
Source: Front Physiol. 2026 Jul 8;17:1813058. doi: 10.3389/fphys.2026.1813058 (PMC13388093; doi:10.3389/fphys.2026.1813058)
Supplement: Supplementary Figure 1 — Assessment of the proportional hazards assumption using Schoenfeld residuals for variables included in the multivariable Cox regression model. [file DataSheet1.pdf]

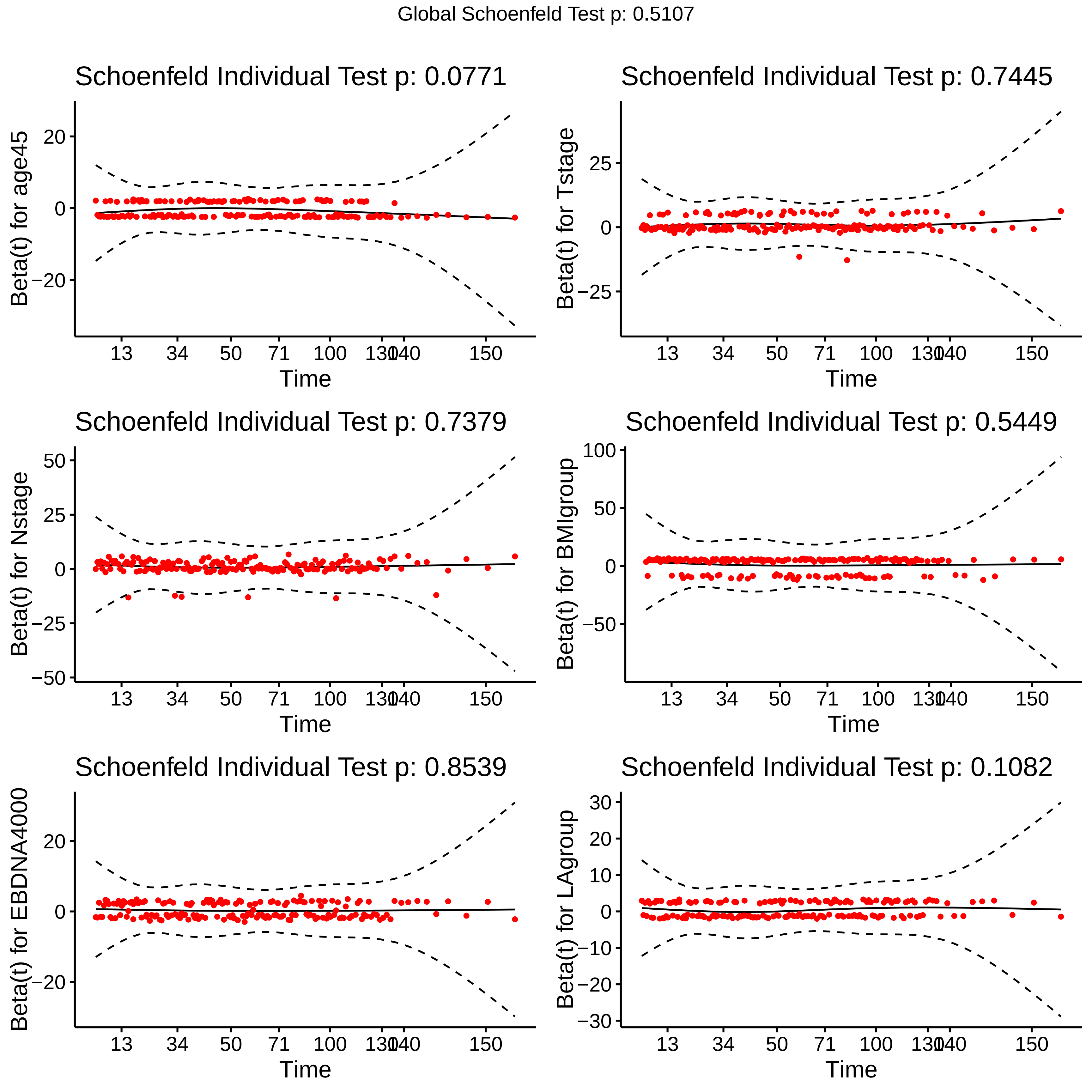

Global Schoenfeld Test p: 0.5107
Schoenfeld Individual Test p: 0.0771
Beta(t) for age45
Time
Schoenfeld Individual Test p: 0.7445
Beta(t) for Tstage
Time
Schoenfeld Individual Test p: 0.7379
Beta(t) for Nstage
Time
Schoenfeld Individual Test p: 0.5449
Beta(t) for BMIgroup
Time
Schoenfeld Individual Test p: 0.8539
Beta(t) for EBDNA4000
Time
Schoenfeld Individual Test p: 0.1082
Beta(t) for LAgroup
Time
13
34
50
71
100
130
140
150
