# Supplementary figures and images for: Integrating the lymphocyte-albumin score into prognostic stratification of nasopharyngeal carcinoma patients treated with concurrent chemoradiotherapy
Source: Front Physiol. 2026 Jul 8;17:1813058. doi: 10.3389/fphys.2026.1813058 (PMC13388093; doi:10.3389/fphys.2026.1813058)

Nomogram Risk — High — Low

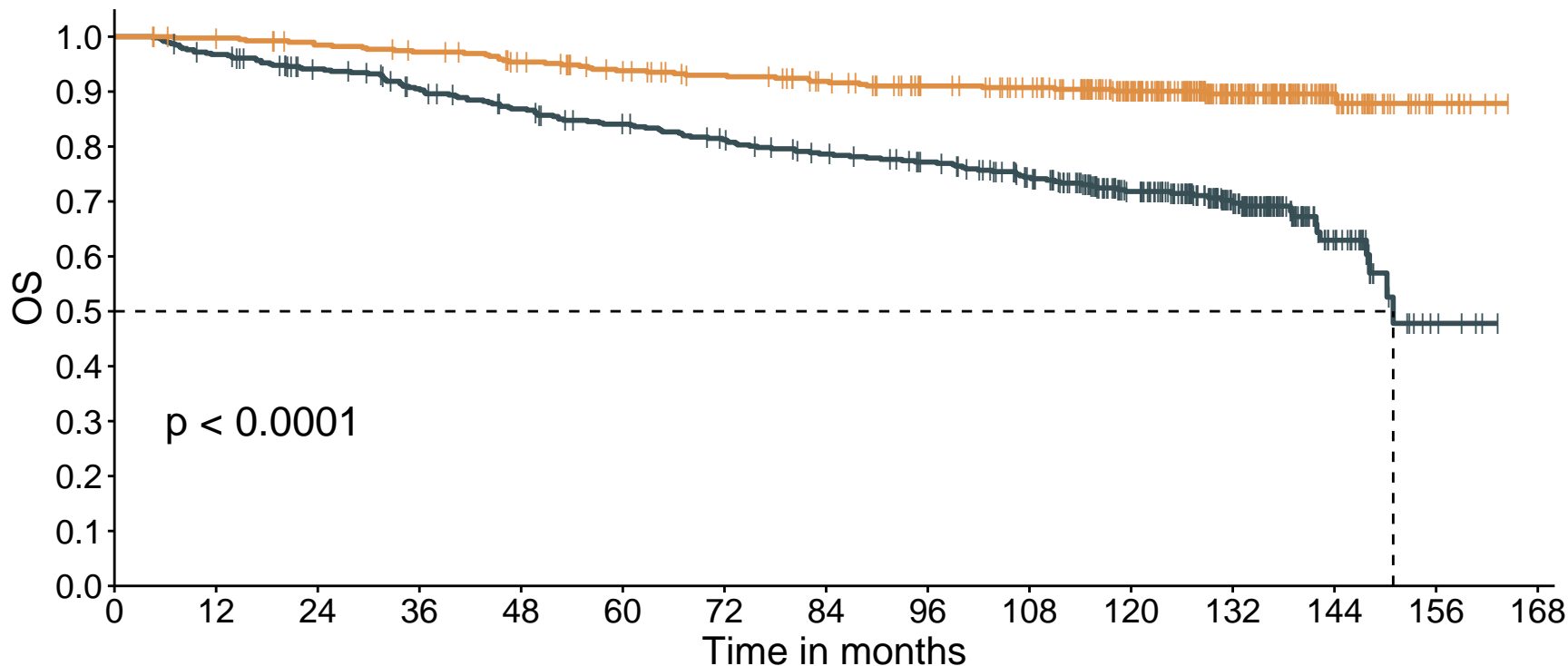

Number at risk

|      |     |     |     |     |     |     |     |     |     |     |     |     |    |   |   |
|------|-----|-----|-----|-----|-----|-----|-----|-----|-----|-----|-----|-----|----|---|---|
| High | 463 | 445 | 421 | 398 | 377 | 359 | 344 | 327 | 313 | 284 | 219 | 138 | 38 | 5 | 0 |
| Low  | 398 | 394 | 385 | 378 | 365 | 351 | 339 | 327 | 309 | 297 | 249 | 150 | 54 | 9 | 0 |

Supplement: Supplementary Figure 2 — Kaplan–Meier curves for overall survival according to nomogram-based risk stratification. [file DataSheet2.pdf]
